# *Caenorhabditis elegans* H1 histone variant HIL-2 is positively regulated by nutrient availability and insulin/IGF signaling but does not affect development

**DOI:** 10.17912/micropub.biology.002228

**Published:** 2026-06-29

**Authors:** Kinsey Fisher, L. Ryan Baugh

**Affiliations:** 1 Department of Biology, Duke University, Durham, NC, United States

## Abstract

&nbsp;&nbsp;&nbsp;&nbsp;&nbsp;&nbsp;&nbsp;&nbsp;&nbsp;&nbsp;&nbsp; Nutrient availability profoundly influences development, and dynamic reorganization of chromatin structure contributes to nutrient-dependent gene regulation. In *Caenorhabditis elegans* , the insulin/IGF-1 signaling (IIS) pathway regulates gene expression to mediate developmental and metabolic responses to nutrient availability. Here, we characterize regulation and function of H1 histone HIL-2 . HIL-2 expression is high in fed larvae and decreases with reduced food availability and decreased IIS activity. We find that *hil-2* does not affect larval development, though *hil-2 ( ok2548 )* displays a larval arrest phenotype. Together with work on another H1 variant, HIL-1 , these results suggest that histone H1 variant dynamics contribute to nutrient-dependent gene regulation.

**Figure 1. hil-2/H1.0 is positively regulated by nutrient availability and IIS but does not affect larval development:** A) Transcript abundance (counts per million) of *hil-2* from RNA-seq of fed and starved (~12 hr after hatch) wild-type worms as well as starved *daf-16 ( mgDf47 ),* *daf-2 ( e1370 )* , and *daf-16 ( mgDf47 ); daf-2 ( e1370 )* mutants (Fisher et al. 2026b). **FDR < 0.01, ***FDR < 0.001, n.s. not significant; Fisher's exact test; 4 biological replicates; error bars reflect standard deviation. B) Nomarski and GFP images of HIL-2 reporter at 1000x magnification for fed (25 mg/ml HB101 ) and starved L1 larvae ~12 hr after hatch. C) Average background-corrected GFP intensity for *hil-2* reporter in fed (25 mg/ml HB101 ) and starved L1 larvae ~12 hr after hatch in wild type, *daf-16 ( mu86 )* and *daf-2 ( e1370 ).* *** P < 0.001, n.s. not significant (one-way ANOVA and *post hoc* Tukey test). Asterisks in legend indicate fed vs. starved within each genotype, while asterisks above data points indicate mutant vs. wild type within a condition. D) Average background-corrected GFP intensity of *hil-2* reporter ~12 hr after hatching in variable densities of food (density of HB101 indicated), as well as starved but supplemented with 0.1% ethanol (EtOH), or complete starvation. *** P < 0.001; n.s. not significant compared to the group with the next highest food density (group to the left) (one-way ANOVA and *post hoc* Tukey test); two biological replicates. A significant difference between 25 mg/ml and 6.3 mg/ml HB101 (P = 0.01) is not indicated. B-D) Identical exposure times used for all genotypes/conditions within a panel. E-G) Length following 48 hr of growth on food. *** P < 0.001, n.s. not significant; t-test on mean of replicates. E) *hil-2 ( ok2548 )* heterozygotes ( *hil-2 ( ok2548 )/ nT1 [ qIs51 ],* wild-type-like, pharyngeal GFP) vs. homozygotes ( *hil-2 ( ok2548 )/ hil-2 ( ok2548 )* , early larval arrest, no GFP). “rf” refers to *hil-2 ( ok2548 )* reduction-of-function allele, and “bal” refers to *nT1 [ qIs51 ]* balancer. F) HIL-2 AID* negative control ( *degron:: hil-2* ) and AID* functional depletion strain with and without 100 μM auxin. G) wild type vs *hil-2* null mutant *syb9670* (or “ *hil-2 ∆”* ).

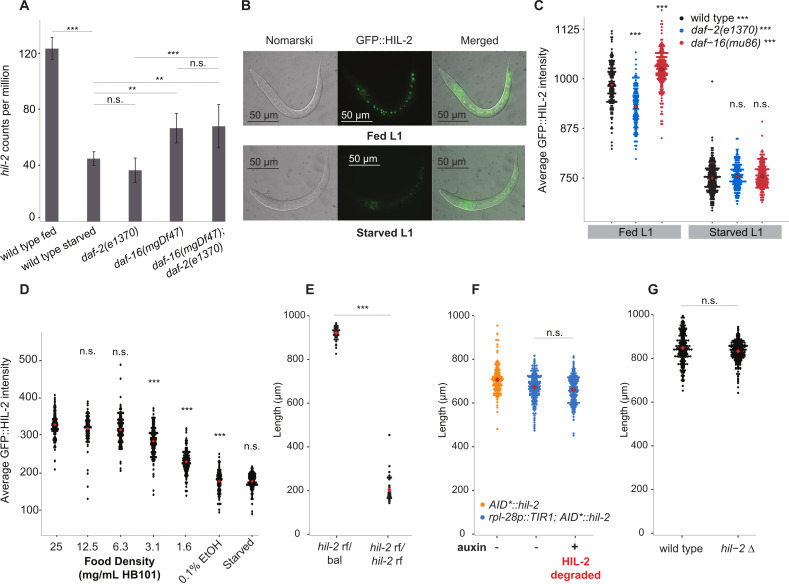



## Description

Animals adjust their physiology to balance developmental progression and survival when faced with different levels of nutrient availability. The nematode *Caenorhabditis elegans* survives on transient microbial food sources in the wild, and it has robust responses to nutrient availability (Baugh and Hu 2020). The widely conserved insulin/IGF-1 signaling (IIS) pathway plays a critical role in matching developmental outcomes to nutritional status in *C. elegans* , including developmental arrest in response to starvation (Baugh 2013; Baugh and Hu 2020; Murphy and Hu 2013). IIS is reduced during starvation, and mutations affecting the sole known insulin/IGF-1 receptor, DAF-2 /InsR, lead to increased starvation resistance, delayed development, and reduced reproductive capacity (Baugh and Sternberg 2006; Gems et al. 1998; Muñoz and Riddle 2003). These effects of *daf-2* on development and particularly starvation resistance are almost entirely dependent on the activation of the forkhead box O transcription factor DAF-16 /FoxO (Fisher et al. 2026b), but the downstream mechanisms through which IIS promotes transcriptional reprogramming and physiological adaptation to nutrient availability remain elusive.

Dynamic reorganization of chromatin appears to mediate nutrient-dependent gene regulation in *C. elegans .* For example, primordial germ cell chromatin undergoes hypercompaction in response to starvation in recently hatched L1 larvae resulting in global transcriptional silencing (Belew et al. 2021). In addition, intestinal chromatin undergoes large-scale reorganization in response to starvation, affecting expression of metabolic and stress-related genes (Al-Refaie et al. 2024).

H1 histones promote chromatin condensation through interactions with linker DNA (Di Liegro et al. 2018), suggesting they may contribute to nutrient-dependent regulation of chromatin organization. Variants from this divergent class of histones are known to have different expression patterns and contribute to specific biological processes (Harshman et al. 2013), but nutritional control of expression and conditional function was only recently reported for an H1 histone. That is, the *C. elegans* genome encodes nine annotated H1 variants (Consortium 2024; Sternberg et al. 2024), and one of them, HIL-1 /H1.0 *,* is transcriptionally up-regulated during starvation via reduced IIS and DAF-16 /FoxO (Fisher et al. 2026a). &nbsp; *hil-1* is a direct target of DAF-16 (Schuster et al. 2010; Tepper et al. 2013), and it promotes resistance to starvation and the bacterial pathogen *Pseudomonas aeruginosa* &nbsp;(Fisher et al. 2026a). Another H1 variant, HIL-2 /H1.0 *,* displays the opposite pattern of regulation from *hil-1* , with *hil-2* mRNA abundance downregulated during starvation in *daf-16-* dependent fashion (Fisher et al. 2026a) ( Fig. 1A ). These observations suggest that IIS transcriptionally regulates activity of specific H1 variants to tune chromatin dynamics in response to nutrient availability.

We used CRISPR-Cas9 to insert GFP at the N-terminus of HIL-2 to visualize HIL-2 expression and validate our RNA-seq results ( Fig. 1A ; Fisher et al. 2026a). GFP:: HIL-2 displayed widespread nuclear expression in fed L1 larvae, with the most distinct expression in epidermal cells and head and tail neurons, and expression was visibly diminished in starved L1 larvae ( Fig. 1B ). We quantified whole-worm GFP:: HIL-2 expression in fed and starved larvae ~12 hr after hatching in wild-type, *daf-16 ( mu86 )* (null), and *daf-2 ( e1370 )* backgrounds *.* GFP:: HIL-2 was expressed at significantly higher levels in fed than starved larvae in all three genotypes ( Fig. 1C ), corroborating RNA-seq ( Fig. 1A ). *daf-2* and *daf-16* had no apparent effect on GFP:: HIL-2 expression in starved larvae, where expression is relatively low ( Fig. 1C ). Although *daf-16 ( mgDf47 )* caused a significant increase in *hil-2* mRNA in starved larvae (Fig 1A), we believe this change was not seen for GFP:: HIL-2 intensity in starved *daf-16 ( mu86 )* mutants ( Fig. 1C ) due to post-transcriptional regulation or a lack of sensitivity in image-based GFP quantification compared to mRNA detection. However, GFP:: HIL-2 expression was decreased and increased by disruption of *daf-2* and *daf-16* , respectively, in fed larvae, consistent with RNA-seq and supporting the conclusion that IIS regulates *hil-2* transcription. However, these results also suggest nutrient-dependent regulation of *hil-2* independent of IIS. GFP:: HIL-2 displayed a dynamic range of expression in response to nutrient availability, with incremental decreases in expression as food density decreased from 6.1 mg/mL *E. coli* HB101 to complete starvation (Fig 1D). Notably, HIL-1 displayed the opposite pattern, with increasing expression as food density decreases (Fisher et al. 2026a). These results suggest that transcription of HIL-2 , like HIL-1 , is regulated by nutrient availability and IIS, but with HIL-2 activated in fed larvae and HIL-1 activated in starved larvae.

Because HIL-2 expression is upregulated in fed larvae by IIS, we were curious if HIL-2 promotes postembryonic development. Notably, *hil-2 ( ok2548 )* (~1700 bp deletion) (Consortium 2012) was annotated as homozygous lethal with an early larval arrest ( *Caenorhabditis* Genetics Center), supporting our hypothesis. We backcrossed *hil-2 ( ok2548 )* and quantified worm length after 48 hr of postembryonic feeding in *hil-2 ( ok2548 )* homozygotes and heterozygotes. The homozygous worms displayed essentially no increase in size in response to feeding (Fig 1E). To validate this phenotype, we used auxin-inducible degradation (AID*) to conditionally degrade HIL-2 by tagging it with a degron. The degron-bearing protein is ubiquitinated by transgenic *Arabidopsis* TIR1 when the plant hormone auxin (or an appropriate analog) is added (Zhang et al. 2015). Successful protein knockdown was confirmed by substantial, though incomplete, GFP degradation, indicating a strong loss-of-function rather than a null. Surprisingly, knockdown of HIL-2 by AID* did not affect postembryonic growth ( Fig. 1F ). Given discrepant results between *ok2548* and AID*, we assayed a full deletion of the *hil-2* coding region, which also had no effect on postembryonic growth ( Fig. 1G ). These results suggest that the larval arrest phenotype observed in *hil-2 ( ok2548 )* is unlikely to result from loss of *hil-2* function but may instead be caused by a linked background mutation. Although *hil-2* did not appreciably affect larval growth, it may be involved in other developmental processes such as reproduction or lifespan.

We show that expression of the H1 variant HIL-2 is governed by nutrient availability and IIS. Interestingly, the H1 paralogs HIL-1 and HIL-2 are both regulated by nutrient availability and IIS, but in opposite patterns, with HIL-1 dramatically upregulated during starvation (Fisher et al. 2026a) and HIL-2 upregulated by feeding (this work). Degradation of all but one H2B variant ( HIS-41 ) occurs in starved L1 larvae, and swapping out H2B variants with HIS-41 during starvation is required for proper execution of the starvation response (Zhu et al. 2023). Distinct H1 histone variants may also be selectively expressed/degraded in response to different environmental conditions to modulate chromatin organization, mediating some of the effects of nutrient availability and IIS on gene regulation. &nbsp;

## Methods

Bacterial strains and preparation

For solid media preparation, one colony of *E. coli* OP50 was added to 100 mL of LB and grown overnight at room temperature then stored at 4°C. Four drops of OP50 were spread onto to 10 cm plates of nematode growth medium (NGM) and plates were incubated at room temperature for 48 hr before being used or stored at 4°C.

For liquid media preparation, one colony of *E. coli* HB101 was added to a 5 mL culture of LB + 50 μg/mL streptomycin and grown overnight at 37°C in a shaking incubator at 180 rpm. This culture was then added to a 1 L culture of TB + 50 μg/mL streptomycin and grown for another 24 hr at 37°C and 180 rpm. Bacteria were centrifuged for 10 min at 4,000 rpm to form a pellet, then were resuspended in S-complete to create a 10x stock (250 mg/mL) that was stored at 4°C. Bacteria was pulled from the 10x stock and added to liquid cultures to achieve desired densities of HB101 .

*C. elegans* maintenance and strains

All *C. elegans* strains were maintained at 20°C on NGM plates seeded with *E. coli* OP50 and passaged by chunking or picking. Strains used in this study are described in the table below. *hil-2 ( ok2548 )* worms were maintained by picking heterozygous, GFP+ L4 larvae and checked for correct segregation of progeny.

*C. elegans* liquid culture

Strains were prepared for bleach to begin experiments as described in *Fisher et al. 2026.* Fed and starved liquid cultures were established by placing 5,000 embryos into 5 ml of S-basal (starved) or S-complete (fed) in 16 mm glass test tubes at 20°C in the dark on a tissue culture roller drum at ~30 rpm. Embryos hatched and arrested as L1 larvae in starvation cultures, and aliquots from these cultures were used for imaging assays.

Quantification of GFP using automated imaging

Aliquots were collected from liquid cultures and washed 3x with S-basal to remove bacteria. ~400 worms were transferred to a 96-well plate with 50 μM sodium azide and imaged using an ImageXpress® Nano automated imager at 100X total magnification under transmitted light and GFP channels. The same exposure time was used for all samples within an experiment. Background intensity was subtracted, and detected objects were manually screened to only include individual, in-focus whole animals. Statistical differences between treatment groups were determined using one-way ANOVA and *post ho* c Tukey tests. Data was visualized using ggplot2 in R.

High-magnification imaging of GFP in L1 larvae

Aliquots were pulled and washed from the liquid cultures as described above. Worms were pelleted and resuspended in 10 mM levamisole in a 1.5 mL Eppendorf tube, then re-pelleted and transferred to a 4% noble agar pad on a microscope slide. Images of worms were taken at 1000X total magnification on a Zeiss AxioImager compound microscope using Nomarski microscopy or a GFP filter with equivalent exposure times. GFP and Nomarski images were merged using Fiji (Schindelin et al. 2012).

Auxin-inducible degradation (AID*)

The synthetic, water-soluble auxin analog (1-naphthaleneacetic acid, potassium salt [K-NAA]) was maintained at a 400 mM stock and stored in the dark at −20°C. 40 mM working stocks were prepared by diluting the auxin in water and storing in the dark at −20°C. To degrade HIL-2 , 100 μM of auxin was added to 10 cm seeded NGM plates and allowed to dry for 1 hr prior to plating embryos. Degradation of HIL-2 was confirmed by dramatic but incomplete loss of GFP signal when observed at 1000x on a Zeiss AxioImager compound microscope.

Length measurements

After synchronization by hypochlorite treatment, ~1000 embryos were plated onto 10 cm NGM plates seeded with a lawn of *E. coli* OP50 and placed at 20°C. After 48 hours, worms were rinsed off the plates using S-basal into 15 mL conical tubes and pelleted at 3,000 rpm for 1 min before being plated onto unseeded 10 cm plates for imaging. For *hil-2 ( ok2548 ),* a GFP filter was used to determine heterozygous vs. homozygous worms. Because the nT1 balancer is marked with GFP, homozygous worms have no GFP and heterozygous worms express pharyngeal GFP. Images were taken on a ZeissDiscovery V20 stereomicroscope at 20x, except for *hil-2 ( ok2548 )* homozygotes, which were imaged at 40x. Worm length was measured using the FIJI WormSizer plugin as previously described (Moore et al. 2013). 3-4 biological replicates were imaged and analyzed for each experiment. T-tests on replicate means were used to determine statistical significance between wild type and *hil-2* perturbation. ggplot2 in R was used to visualize data.

## Reagents

**Table:** 

**Strain**	**Genotype**	**Description**	**Source**
N2	Wild type	Sternberg N2	Paul Sternberg – California Institute of Technology
LRB621	*hil-2 ( ok2548 )/ nT1 [ qIs51 ] *	Deletion (~1700 bp)	CGC &nbsp; Backcrossed 4x to *nT1 [ qIs51 ]* balancer
PHX9670	*hil-2 (syb9670) *	Full deletion of *hil-2* using CRISPR	Generated in this work by SunyBiotech
PHX6763	hil-2 ( syb6763 ) [GFP::linker::AID*:: * linker::3xFLAG::linker:: hil-2 ] *	GFP, AID* and 3xFLAG tagged to endogenous *hil-2* N-terminus with CRISPR	Generated in this work by SunyBiotech
LRB573	*daf-2 ( e1370 ); hil-2 ( syb6763 ) * &nbsp;	*hil-2 ( syb6763 )* crossed to *daf-2 ( e1370 ) *	Generated in this work
LRB574	*daf-16 ( mu86 ); hil-2 ( syb6763 ) * &nbsp;	*hil-2 ( syb6763 )* crossed to *daf-16 ( mu86 ) *	Generated in this work
LRB569	* keaSi10 [rpl-28p::TIR1::mRuby:: unc-54 3'UTR + Cbr-unc-119 (+)]; hil-2 ( syb6763 ) *	*hil-2 ( syb6763 )* with ubiquitous TIR1 expression	Generated in this work

**Table 1. Strains used in this study.**
